# Genomic Analysis and Comparison of Two Gonorrhea Outbreaks

**DOI:** 10.1128/mBio.00525-16

**Published:** 2016-06-28

**Authors:** Xavier Didelot, Janina Dordel, Lilith K. Whittles, Caitlin Collins, Nicole Bilek, Cynthia J. Bishop, Peter J. White, David M. Aanensen, Julian Parkhill, Stephen D. Bentley, Brian G. Spratt, Simon R. Harris

**Affiliations:** a Department of Infectious Disease Epidemiology, Imperial College London, London, United Kingdom; b Wellcome Trust Sanger Institute, Wellcome Trust Genome Campus, Hinxton, Cambridgeshire, United Kingdom; c Department of Biology, Drexel University, Philadelphia, Pennsylvania, USA; d South African Tuberculosis Vaccine Initiative, Institute of Infectious Disease and Molecular Medicine, University of Cape Town, Cape Town, South Africa; e International AIDS Vaccine Initiative, Human Immunology Laboratory, Imperial College London, London, United Kingdom; f Modelling and Economics Unit, Centre for Infectious Disease Surveillance and Control, Public Health England, London, United Kingdom; g MRC Centre for Outbreak Analysis and Modelling, School of Public Health, Imperial College London, London, United Kingdom; h NIHR Health Protection Research Unit in Modelling Methodology, School of Public Health, Imperial College London, London, United Kingdom; i Centre for Genomic Pathogen Surveillance, Wellcome Trust Genome Campus, Hinxton, Cambridgeshire, United Kingdom

## Abstract

Gonorrhea is a sexually transmitted disease causing growing concern, with a substantial increase in reported incidence over the past few years in the United Kingdom and rising levels of resistance to a wide range of antibiotics. Understanding its epidemiology is therefore of major biomedical importance, not only on a population scale but also at the level of direct transmission. However, the molecular typing techniques traditionally used for gonorrhea infections do not provide sufficient resolution to investigate such fine-scale patterns. Here we sequenced the genomes of 237 isolates from two local collections of isolates from Sheffield and London, each of which was resolved into a single type using traditional methods. The two data sets were selected to have different epidemiological properties: the Sheffield data were collected over 6 years from a predominantly heterosexual population, whereas the London data were gathered within half a year and strongly associated with men who have sex with men. Based on contact tracing information between individuals in Sheffield, we found that transmission is associated with a median time to most recent common ancestor of 3.4 months, with an upper bound of 8 months, which we used as a criterion to identify likely transmission links in both data sets. In London, we found that transmission happened predominantly between individuals of similar age, sexual orientation, and location and also with the same HIV serostatus, which may reflect serosorting and associated risk behaviors. Comparison of the two data sets suggests that the London epidemic involved about ten times more cases than the Sheffield outbreak.

## IMPORTANCE

The recent increases in gonorrhea incidence and antibiotic resistance are cause for public health concern. Successful intervention requires a better understanding of transmission patterns, which is not uncovered by traditional molecular epidemiology techniques. Here we studied two outbreaks that took place in Sheffield and London, United Kingdom. We show that whole-genome sequencing provides the resolution to investigate direct gonorrhea transmission between infected individuals. Combining genome sequencing with rich epidemiological information about infected individuals reveals the importance of several transmission routes and risk factors, which can be used to design better control measures.

## INTRODUCTION

Gonorrhea is a sexually transmitted disease (STD) caused by the bacterium *Neisseria gonorrhoeae*. In the United Kingdom, gonorrhea is one of the most common bacterial STDs, and its reported incidence has markedly increased since 2008 in both men and women, reaching a total of 35,000 diagnosed cases in 2014 (1). Treatment with antimicrobials is usually successful, but increased resistance to many frontline antibiotics has recently been observed (2, 3). Implementation of effective control measures to mitigate the spread of gonorrhea is difficult due to a lack of understanding of the importance of the complex transmission routes and reservoirs (4). Traditional epidemiological studies are complicated by the facts that gonorrhea can be carried asymptomatically for months in about 10% of men and 50% of women (5) and in the United Kingdom infects disproportionately men who have sex with men (MSM) and young heterosexuals of black ethnicity and features significant geographical and temporal variations (6).

Molecular epidemiology approaches—for example, *opa* typing (7), *N. gonorrhoeae* multiantigen sequence typing (NG-MAST) (8), or multilocus sequence typing (MLST) (9)—have proved helpful to demonstrate that multiple strains often circulate simultaneously within a host population, to detect emerging, often resistant, clones, and to identify clusters of individuals infected with the same strain. Primarily, these typing schemes are useful to rule out transmission links between individuals who carry different types and do not have sufficient resolution to shed light on fine patterns of transmission between individuals carrying the same type.

In recent years, bacterial epidemiology has started to be transformed by the availability of fast, affordable whole-genome sequencing, which can help identify transmission links sometimes even at the level of direct transmission between individuals (10–12). The value of genomic data to investigate local outbreaks has been demonstrated for several bacterial pathogens, including *Staphylococcus aureus* (13, 14), *Clostridium difficile* (15, 16), and *Mycobacterium tuberculosis* (17, 18). In a study of gonococcal epidemiology, whole-genome sequencing has recently been applied to a collection of isolates from across the United States, showing important structuring of the pathogen population with both geography and sexual orientation of the hosts (19).

To test the usefulness of gonococcal genome sequencing to track transmission at the finer scale of outbreaks occurring within a city, we selected two local isolate collections with different epidemiological properties, both of which have been previously described without genomic data. The Sheffield isolate collection was assembled between 1995 and 2000 at the single genitourinary clinic in the city, from a mostly heterosexual population in which contact tracing was performed. Multiple strains of the pathogen have been found to circulate using *opa* typing (20, 21) and NG-MAST typing (22), and in both cases molecular typing was correlated with known sexual contact links. We applied whole-genome sequencing to 132 isolates with the most prevalent NG-MAST type, ST12.

The London collection consists of 2,045 isolates sampled between June and November 2004 from 13 major sexual health clinics throughout London, representing 54% of the 3,754 cases reported in London at that time (23, 24). Contact tracing information was not available, but rich metadata were recorded for each infected individual, including ethnic background, HIV status where known, postcode, and reporting clinic. All isolates were previously typed using NG-MAST, revealing the coexistence of a large number of strains, some of which were mostly MSM associated and others mostly heterosexual associated (23). Forty-five percent of infected individuals had one of 21 major strains, among which NG-MAST type ST225 was the most strongly MSM associated (with 92% of cases being MSM) and not geographically clustered within London (24). We applied whole-genome sequencing to 105 isolates of ST225.

Our aim was to assess the potential benefits that whole-genome sequencing data could provide for epidemiology within single NG-MAST types. A relatively recent history of transmission would be expected due to the shared type and the localized sampling frames. The epidemiological backgrounds of the two studies are very different, with the Sheffield data set coming from a single STD clinic in a city more than 10 times smaller than the London data set, which was collected at 13 clinics. Furthermore, the Sheffield isolates are heterosexual associated, whereas the London isolates are MSM associated. Consequently, this will also allow us to investigate how differences in variation within the two data sets reflect their underlying context.

## RESULTS AND DISCUSSION

### Genomic analysis of the Sheffield data set.

We sequenced the genomes of 132 isolates from Sheffield dated from 1995 to 2000, all of which had the same genotype, ST12, as defined by NG-MAST (see Table S1 in the supplemental material). Few recombination events were detected using Gubbins (25), with a relative effect of recombination compared to mutation of *r*/*m* = 0.04, which was much lower than those of previous reports based on species-wide diversity (19, 26). This may reflect a difference in the effect of recombination when measured at different scales or diversity or between different lineages, as previously reported in other bacterial pathogens, such as *Staphylococcus aureus* (27), *Clostridium difficile* (15), or *Streptococcus pneumoniae* (28). The few detected recombination events occurred mainly in genes coding for outer membrane proteins that undergo antigenic variation and have previously been described as highly recombinant—for example, *opa* and *pil* genes (29). After both repetitive and recombinant regions were removed, a total of 156 variable sites were found to distinguish the genomes from each other. A maximum likelihood phylogeny was constructed using these data and showed a strong temporal signal based on the correlation between root-to-tip distances and isolation dates for all leaves in the phylogeny (*R*² = 0.49) (see Fig. S1 in the supplemental material).

**FIG 1:**
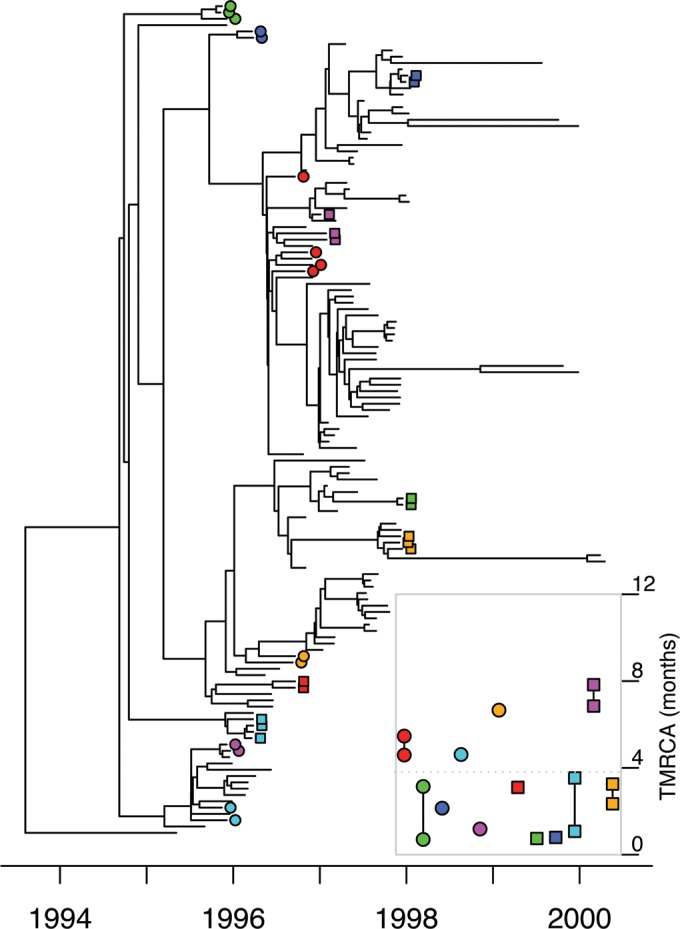
Timed phylogeny for the Sheffield data set reconstructed using BEAST. Known sexual contacts are indicated by uniquely colored circles and squares. (Inset) Intervals for the time to the last common ancestor (TMRCA) of each pair within a group of known sexual contacts.

We therefore applied the Bayesian evolutionary analysis software BEAST (30) to these data in order to reconstruct a timed phylogeny (Fig. 1). The molecular clock rate was estimated to be 1.41 × 10^−6^ single nucleotide polymorphisms (SNPs) per site per year with a 95% confidence interval (CI) of 1.15 × 10^−6^ to 1.70 × 10^−6^. This rate was equivalent to 3.05 (95% CI, 2.47 to 3.67) mutations per year across the genome, is in good agreement with previous estimates in *N. gonorrhoeae* (19, 26) and the related species *Neisseria meningitidis* (31), and was around the middle of the range of reported values for other bacterial pathogens (32, 33).

Known sexual contacts were significantly clustered on the timed phylogeny (Fig. 1 [permutation test]; see Fig. S2 in the supplemental material [*P* < 10^−4^]). For the 25 pairs of known sexual contacts (see Table S1 in the supplemental material), the median time to the most recent common ancestor (TMRCA) of the isolated bacteria was 3.4 months, with an interquartile range from 2.3 to 5.1 months (Fig. 1, inset). When considering direct transmission between individuals sampled at roughly the same time, with no diversity being transmitted (due to a strong transmission bottleneck), the most recent common ancestor of the two sampled genomes would have existed within the pathogen population of the infector (34–36). The TMRCA is therefore a lower bound for the time from infection to sampling of the donor and an upper bound for the time from infection to sampling of the recipient, so that the mean TMRCA for pairs of contacts is expected to be approximately equal to the average duration of infection. Our result is in good agreement with previous estimates ranging from 2 to 6 months for the average duration of gonorrhea infection in several modeling studies (37–39). This duration is significantly longer than the few days reported for the incubation period in male experimental challenges (40), even accounting for the few additional days taken from symptom onset to care seeking (41). However, since the Sheffield population is predominantly heterosexual, the mean duration of infection is an average between men and women and takes into account the fact that a large fraction of women as well as a smaller proportion of men can remain asymptomatic for extended periods of time (42).

Since all pairs of known sexual contacts have a TMRCA of less than 8 months, we decided to use this value as the maximum threshold for the TMRCA between two individuals who have directly infected each other. This threshold should conservatively rule out transmission for pairs of genomes with a higher TMRCA, because all pairs of known sexual contacts fulfill this criterion, and yet not all of them are direct transmission pairs since the data include four triplets and one quadruplet of sexual contacts (Fig. 1, inset). Pairs of genomes sampled more than 8 months apart are unlikely to be transmission links. We compared all pairs of genomes sampled within 3 months of each other, which applies to all 25 pairs of known sexual contacts, and found that 24% (398/1,632) had a TMRCA of less than 8 months, including the known sexual contacts (see Fig. S3 in the supplemental material). Since all of these pairs were sampled within a short period of time in the same city and had the same NG-MAST type, a nongenomic analysis could not rule out transmission for any of them, but with the help of genomic data, we can confidently rule out transmission for the majority.

**FIG 2:**
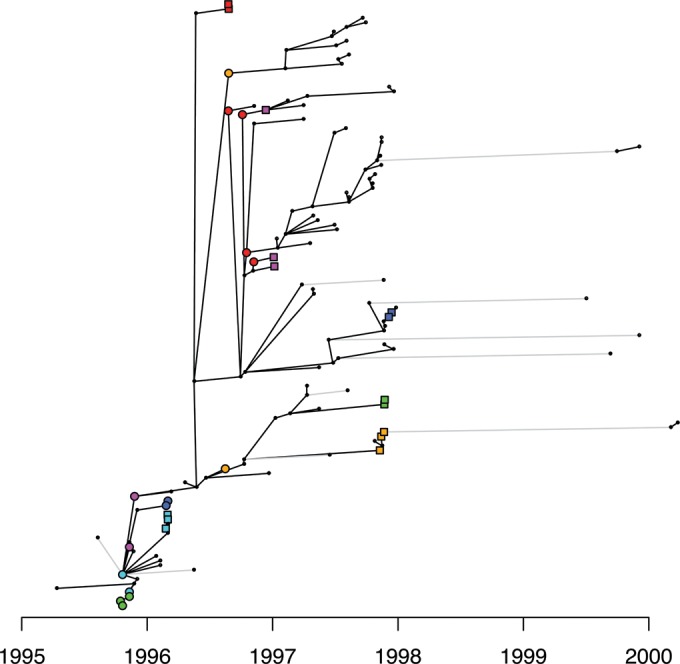
Transmission tree for the Sheffield data set reconstructed using Outbreaker. Cases are indicated by black dots, except for known sexual contacts, who are indicated using the same markers as in Fig. 1. Each case is aligned on the *x* axis with its reporting date, and the *y* axis is arbitrary. Black links between cases indicate inferred direct transmission, and gray links indicate indirect transmission through at least one unsampled case.

To complement the phylogenetic analysis of the Sheffield data, we applied Outbreaker, which allows the direct reconstruction of a transmission tree representing transmission pathways within a sample, including the possibility of unsampled missing links in the transmission chains (43). Known sexual contacts clustered together on the reconstructed transmission tree, with at most three intermediates in the transmission chain between contacts (Fig. 2). When looking at all pairs of individuals in the Sheffield data set sampled within 3 months of each other, a strong correlation was found between their TMRCA in the BEAST tree and whether or not Outbreaker inferred them to be linked (see Fig. S4 in the supplemental material) (Kruskal-Wallis test [KWT], *P* < 10^−15^; mean TMRCA of 0.28 and 1.54 years for pairs linked and unlinked by Outbreaker, respectively), indicating a good agreement between the two approaches.

### Genomic analysis of the London data set.

We sequenced the genomes of 105 isolates sampled in London between June and November 2004, all of which were representatives of NG-MAST type ST225 (see Table S2 in the supplemental material). Significant recombination was detected using Gubbins (25), with *r*/*m* = 1.17, which was higher and in better agreement with previous reports (19, 26) than the value for the Sheffield data. Most of the recombination events occurred on deep branches and mainly affected outer membrane and pilus genes, repeat regions, and a prophage. A total of 167 variable sites were found after repetitive and recombinant regions were removed.

**FIG 3:**
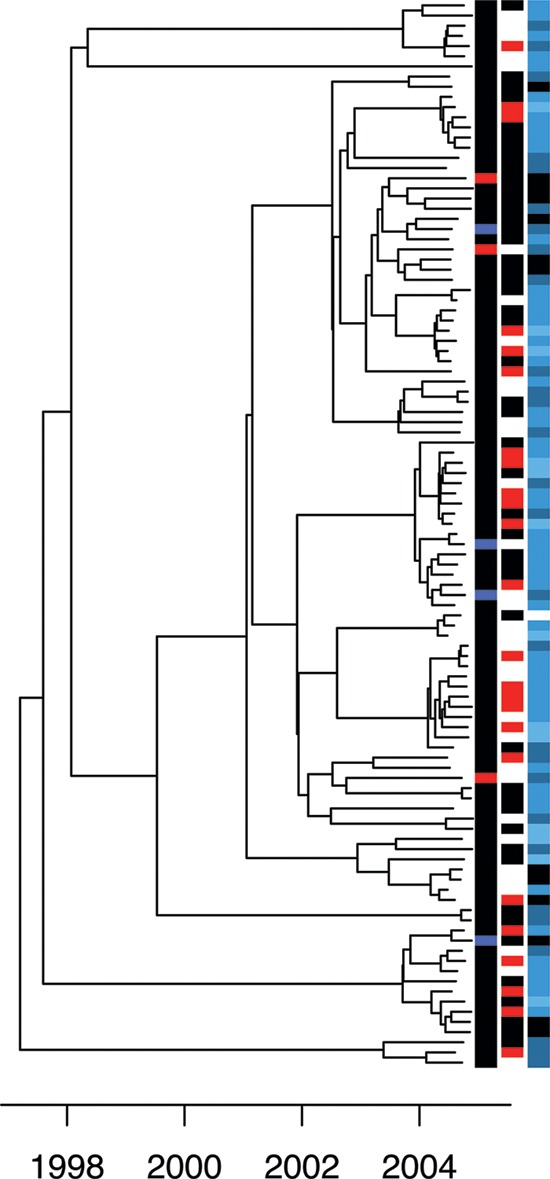
Timed phylogeny for the London data set reconstructed using BEAST. Each isolate is annotated on the right-hand side as follows. First column: black for MSM, blue for heterosexual men, and red for heterosexual women. Second column: black for HIV negative and red for HIV positive. Third column: number of reported United Kingdom partners in the last 3 months, with black for zero, gray for one, dark blue for two to five, and light blue for six or more.

The temporal signal was weak in this data set due to the short sampling span of only 6 months, so that the molecular clock rate could not be directly estimated with confidence from this data set. The distribution of root-to-tip distance versus sampling dates was, however, compatible with that expected under the rate estimated for the Sheffield data set (see Fig. S5 in the supplemental material). A timed tree was therefore produced using BEAST (30), but forcing the molecular clock rate to be equal to that estimated for the Sheffield data (Fig. 3). We also tried to apply Outbreaker to this data set as we did for the Sheffield data set, but the results were not biologically meaningful due to the shorter sampling interval for the London data, which is incompatible with Outbreaker’s assumption of a constant sampling density from beginning to end of an outbreak (43).

The analysis of the Sheffield data suggested a threshold of at most 8 months for the TMRCA compatible with direct transmission from one individual to another. The London data come from a predominantly MSM population, but the same threshold should be applicable since the asymptomatic frequency of anorectal cases among MSM is ~80% (44), similar to the ~75% in heterosexual females (42). We therefore applied the same criterion to all pairs of individuals in the London data who were sampled within 3 months of each other. We found that transmission was a possibility for 4% (165/4,251) of such pairs (see Fig. S6 in the supplemental material). Likely transmission links were significantly associated with shorter geographical distances between postcodes of residence in London (KWT, *P* = 6 × 10^−3^; mean, 10.3 versus 19.1 km), as would be expected if sexual partnerships tend to be geographically clustered. The age difference was significantly lower for linked individuals than for unlinked individuals (KWT, *P* = 1.3 × 10^−4^; mean, 7.5 versus 9.8 years), likely reflecting a tendency for sexual partners to be about the same age, as found by behavioral surveys such as Natsal-3 (the third of the National Surveys of Sexual Attitudes and Lifestyles) (45). Linked individuals reported the same sexual orientation more often than expected by chance for this sample of individuals (Fisher’s exact test [FET], *P* = 6 × 10^−3^). This significant similarity in location, age, and sexuality of linked individuals suggests that the transmission analysis, which was based only on pathogen genome similarity, successfully captured the correct transmission links. Among the 105 individuals, 24 were HIV positive, 47 were HIV negative, and 33 had unknown HIV status. Relative to the number of transmission links involving an HIV-positive individual and an HIV-negative individual, there was an excess of transmission links found between pairs of individuals who were both HIV positive (FET, *P* = 2.6 × 10^−3^), and simultaneously there was a dearth of transmission links when both individuals were HIV negative (FET, *P* = 0.03).

Among the 105 individuals in the London sample, the genomic analysis above suggested that 29 did not have any likely transmission link, 19 had one, 8 had two, 14 had three, and the remaining 35 had between four and eleven links. This number of likely transmission links for a given individual was not significantly associated with sexual orientation (KWT, *P* = 0.13; mean, 3.19 versus 1.28 links), reported sex abroad (KWT, *P* = 0.41; mean, 2.37 versus 3.41 links), or reported previous gonorrhea (KWT, *P* = 0.33; mean, 3.52 versus 2.89 links) but was significantly increased for individuals who were HIV positive (KWT, *P* = 1.6 × 10^−4^; mean, 5.25 versus 2.08 links), in accordance with the observation above of a higher number of links between HIV-positive individuals. This observation may indicate a role of HIV infection in susceptibility and transmissibility of gonorrhea (46, 47), may be caused by both HIV and gonorrhea infection being linked with the same high-risk behaviors, or both. It is also probable that HIV-positive MSM have on average more partners and engage in serosorting and riskier behaviors (48, 49). A significant correlation was found between the number of transmission links and the reported number of sexual partners in the United Kingdom in the last 3 months (Spearman’s rank correlation test, rho = 0.4; *P* = 2 × 10^−5^). This correlation was partly explained by a higher number of reported partners for HIV-positive individuals (KWT, *P* = 2.2 × 10^−3^; mean, 8.03 versus 2.61), but a correlation was also suggested between number of links and number of partners when analyzing HIV-positive and HIV-negative individuals separately (rho = 0.5, *P* = 0.01, and rho = 0.3, *P* = 0.04, respectively).

### Comparison between the Sheffield and London data sets.

**FIG 4:**
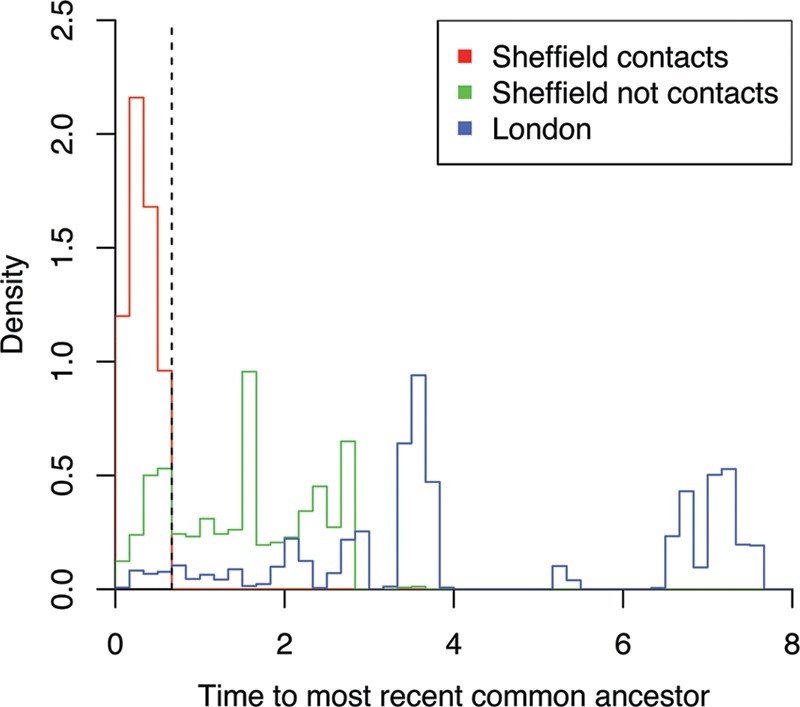
Density histograms of the time to the most recent common ancestor for all pairs of cases sampled within 3 months of each other. The Sheffield data are shown in red when a sexual contact was reported and in green otherwise, whereas the London data are shown in blue.

Having analyzed the Sheffield and London data sets separately, we now turn to comparative analysis between them. The temporal sampling frames are different in the two data sets since sampling in Sheffield happened over 6 years, whereas the London data cover only 6 months. It is therefore not possible, for example, to compare the frequencies with which a putative transmission donor is found for cases in both data sets, because this frequency would be expected to be smaller in London, since donors would more often have been reported before sampling started. However, direct comparisons become possible by focusing on pairs of isolates sampled at approximately the same time, since the relationships between such pairs should not be affected by differences in sampling frame duration. Having reconstructed dated phylogenies for both data sets (Fig. 1 and 3), we can compare the distributions of TMRCA for all pairs of isolates sampled within 3 months of each other (Fig. 4). As previously noted, a larger proportion of pairs have a TMRCA under 8 months in Sheffield relative to London (24% versus 4%), and overall the London pairs have an average TMRCA of 4.4 years compared to 1.5 years for the Sheffield pairs, with the difference being highly significant (KWT, *P* < 1 × 10^−15^). More pairs of individuals in Sheffield are therefore likely to have infected each other compared to in London, and this result is robust to the choice of other TMRCA thresholds than 8 months for when to rule out transmission (Fig. 4).

This difference between the Sheffield and London data sets can be explained from a population genetics viewpoint by a pathogen effective population size (*N*_e_) roughly three times higher in London relative to Sheffield (50). Using a metapopulation analogy, the effective population size of a pathogen can be shown to be proportional to the number of infected individuals and inversely proportional to the transmission rate (51–53). A recent study estimated that gonorrhea spreads two to three times faster in MSM than heterosexual networks (54), which is consistent with a lower proportion of asymptomatic cases in men compared to women and symptoms that are more likely to result in rapid care seeking (5, 42). This difference should contribute to a lower *N*_e_, suggesting that the number of infected individuals in London at a given time may have been six to nine times higher than in Sheffield for the two specific lineages of gonorrhea studied here, namely, NG-MAST ST12 in Sheffield and ST225 in London. This estimation is in broad agreement with the similar number of reported cases in Sheffield and London, despite the sampling interval being about ten times longer in the former.

### Conclusions.

We have presented the first genomic epidemiology investigation of two large, localized outbreaks of gonorrhea—one including 132 isolates from Sheffield collected over 6 years among a mostly heterosexual population, and the other including 105 isolates from London gathered over half a year from a mainly MSM population. We showed that whole-genome sequencing can be used to predict person-to-person transmission events, which we combined with epidemiological information about the infected individuals to reveal patterns of transmission and infection risk factors. Importantly, both isolate collections had previously been assigned to single NG-MAST types, highlighting again the superior resolution provided by genomic data for molecular epidemiology. Pairs of cases detected in the same town, within 3 months of each other and carrying the same molecular type, would be considered to be “linked” using traditional molecular epidemiology, and yet we showed that genome sequencing ruled out transmission for the majority of such pairs in both outbreaks. Furthermore, the proportions of pairs of cases for which transmission were found to be likely using genomic comparisons were very different in Sheffield (24%) and in London (4%), and we estimated that the number of cases was six to nine times higher in London compared to Sheffield. Molecular typing using NG-MAST remains useful to identify cases that are part of the same local outbreak, but our results clearly show that a type does not represent a uniform epidemiological entity. As cost effectiveness and turnaround time of whole-genome sequencing continue to improve, it will become an increasingly important tool in the investigation and control of gonococcal outbreaks, but unlocking its full potential requires the simultaneous application of traditional epidemiological techniques such as the use of questionnaires or contact tracing.

## MATERIALS AND METHODS

### Bacterial isolates.

From the previously described Sheffield collection (20–22), which was sampled between 1995 and 2000, we included 132 out of 140 isolates of the most prevalent NG-MAST type, ST12. Table S1 in the supplemental material contains the list of these isolates and associated metadata. From the previously described London collection (23, 24), which consists of 2,045 isolates sampled between June and November 2004, we included 105 out of 124 isolates from the most MSM-associated NG-MAST type, ST225. Table S2 contains the list of these isolates and associated metadata. Exclusion of 8 and 19 isolates from the Sheffield and London collections, respectively, was due to loss of samples, loss of associated metadata, or failure to grow or sequence.

### Whole-genome sequencing.

For each of the two data sets, the oldest available isolate was used for the production of a reference genome using 454. A 3-kb library was prepared and sequenced on a 1/4 plate run. For the London reference isolate, this produced 139.2 Mbp. *De novo* assembly was carried out using Newbler, resulting in 12 scaffolds with a total length of 2.14 Mbp. The sequencing of the Sheffield isolate produced 91.1 Mbp and 13 scaffolds with a total length of 2.14 Mbp. The length of these two reference genomes is in good agreement with previous reports of the lengths of *N. gonorrhoeae* genomes (19, 26, 55). Both assemblies were annotated using the RAST annotation pipeline (56) and manually curated. Prophages were detected using the online tool PHAST (57). Further mobile genetic elements were detected through genome comparisons with already published *N. gonorrhoeae* genomes and BLAST analysis. Repeats were detected using the EMBOSS tools einverted and equicktandem (58).

The genomes of all Sheffield isolates and all London isolates were sequenced on an Illumina HiSeq2000 with 100-bp paired-end reads. Paired-end reads were mapped against the corresponding reference genome using SMALTv0.7.5 (http://www.sanger.ac.uk/science/tools/smalt-0) with subsequent realignment around indels using GATKv1.5.9 (59). Single nucleotide polymorphisms (SNPs) were called as previously described (60). Recombination was detected using Gubbins (25), and recombinant SNPs were excluded. Furthermore, SNPs within mobile genetic elements as well as repetitive regions were also excluded. This resulted in alignments containing 156 and 167 SNPs for Sheffield and London, respectively.

### Phylogenetic analysis.

Maximum likelihood phylogenies were constructed using phyml (61). In the Sheffield tree, we included a single genome from London, and in the London tree, we included a single genome from Sheffield, to be used as outgroups in order to root the trees. Comparison of root-to-tip distances with isolation dates for each genome revealed a strong temporal signal in the Sheffield data (see Fig. S1 in the supplemental material) but not in London (see Fig. S5 in the supplemental material) due to a short sampling interval. The Bayesian evolutionary analysis software BEAST (30) was applied to both data sets, with the molecular clock of the London data set being forced equal to the rate estimated for the Sheffield data set. The resulting timed phylogenies are shown in Fig. 1 and 3. We used BEAST version 1.8.2 with the default HKY substitution model, the default coalescent model with constant population size, and a strict clock model with rate prior distribution Exponential(1) for the Sheffield data and fixed rate for the London data. For each of the two data sets, we performed four runs of 10^7^ iterations, which were compared in Tracer (http://beast.bio.ed.ac.uk/tracer) to confirm convergence.

### Transmission analysis.

We sought a value for the threshold on the time to the most recent common ancestry of two genomes beyond which direct transmission between the two corresponding individuals can be discounted (15). Based on known sexual contacts in the Sheffield data set, we estimated that 8 months was an appropriate value for this threshold. We applied this criterion to all pairs of genomes sampled within 3 months of each other in both Sheffield and London to determine for each pair whether transmission was likely or not. Pairs of genomes for which transmission was likely are shown in Fig. S3 and S6 in the supplemental material for the Sheffield and London data sets, respectively. We also used Outbreaker, a Bayesian approach for reconstructing a transmission tree from dated genetic data (43). For the generation time, we used a discretized gamma distribution with a mean of 90 days and a standard deviation of 40 days, which has high variance reflecting our lack of knowledge of exact values (62). The Outbreaker output for the Sheffield data is shown in Fig. 2 and compared with the BEAST-based approach in Fig. S4 in the supplemental material. Outbreaker did not produce meaningful results for the London data due to the short sampling frame, which implies that the donor of many cases would have occurred and been reported before the sampling frame.

### Nucleotide sequence accession numbers.

Sequence data have been deposited in the European Nucleotide Archive. Accession numbers for the Illumina data from each isolate can be found in Tables S1 and S2 in the supplemental material.

## SUPPLEMENTAL MATERIAL

Figure S1  (A) Maximum likelihood tree reconstructed for the Sheffield genomes. (B) Temporal signal in the tree. The signal was strong enough to estimate the evolutionary rate shown by the solid line. Download Figure S1, PDF file, 0.01 MB

Figure S2  Permutation test comparing the mean TMRCA for known sexual contacts (in red) with values that would be obtained if the sexual contact labels were permuted at random (histogram). Download Figure S2, PDF file, 0 MB

Figure S3  Links between the Sheffield genomes, based on the 8-month maximum TMRCA criterion. Download Figure S3, PDF file, 0.1 MB

Figure S4  Distribution of TMRCA between pairs of genomes in the Sheffield data set that have been assessed to be directly linked by Outbreaker (red) or not (blue). Download Figure S4, PDF file, 0 MB

Figure S5  (A) Maximum likelihood tree reconstructed for the London genomes. (B) Temporal signal in the tree. The signal was not strong enough to estimate the evolutionary rate, and instead the solid line represents the rate estimated in Fig. S1 based on the Sheffield data. Download Figure S5, PDF file, 0.01 MB

Figure S6  Links between the London genomes, based on the 8-month maximum TMRCA criterion. Download Figure S6, PDF file, 0.01 MB

Table S1  List of 132 genomes in the Sheffield data set. Table S1, PDF file, 0.03 MB

Table S2  List of 105 genomes in the London data set. Table S2, PDF file, 0.04 MB
