# Supplementary figures and images for: Genomic Analysis and Comparison of Two Gonorrhea Outbreaks
Source: mBio. 2016 Jun 28;7(3):e00525-16. doi: 10.1128/mBio.00525-16 (PMC4937209; doi:10.1128/mBio.00525-16)

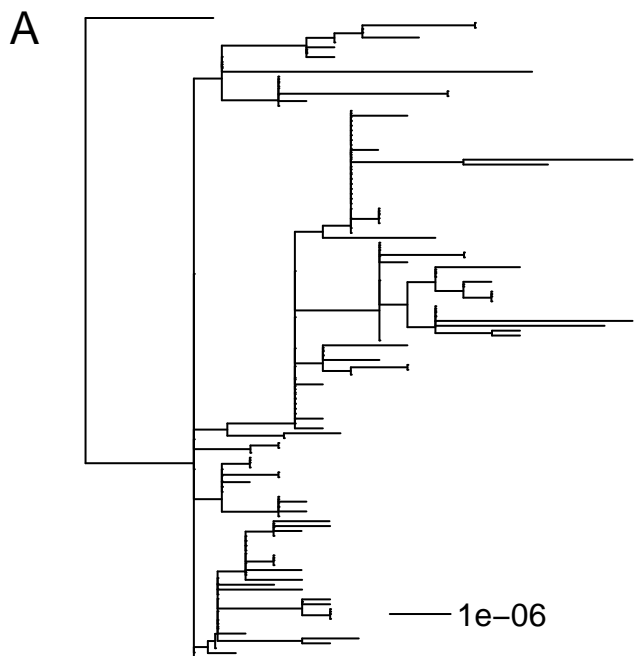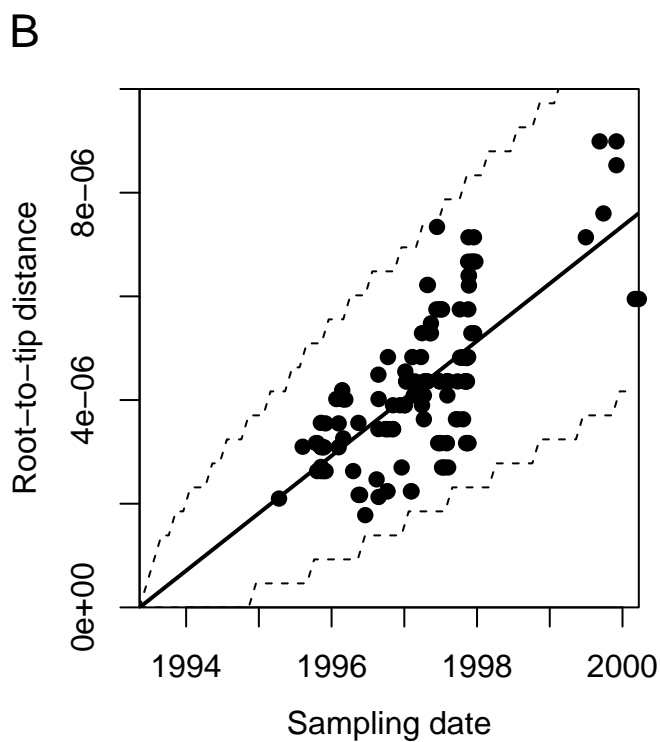

Supplement: Figure S1 — (A) Maximum likelihood tree reconstructed for the Sheffield genomes. (B) Temporal signal in the tree. The signal was strong enough to estimate the evolutionary rate shown by the solid line. Download [file mbo003162861sf1.pdf]

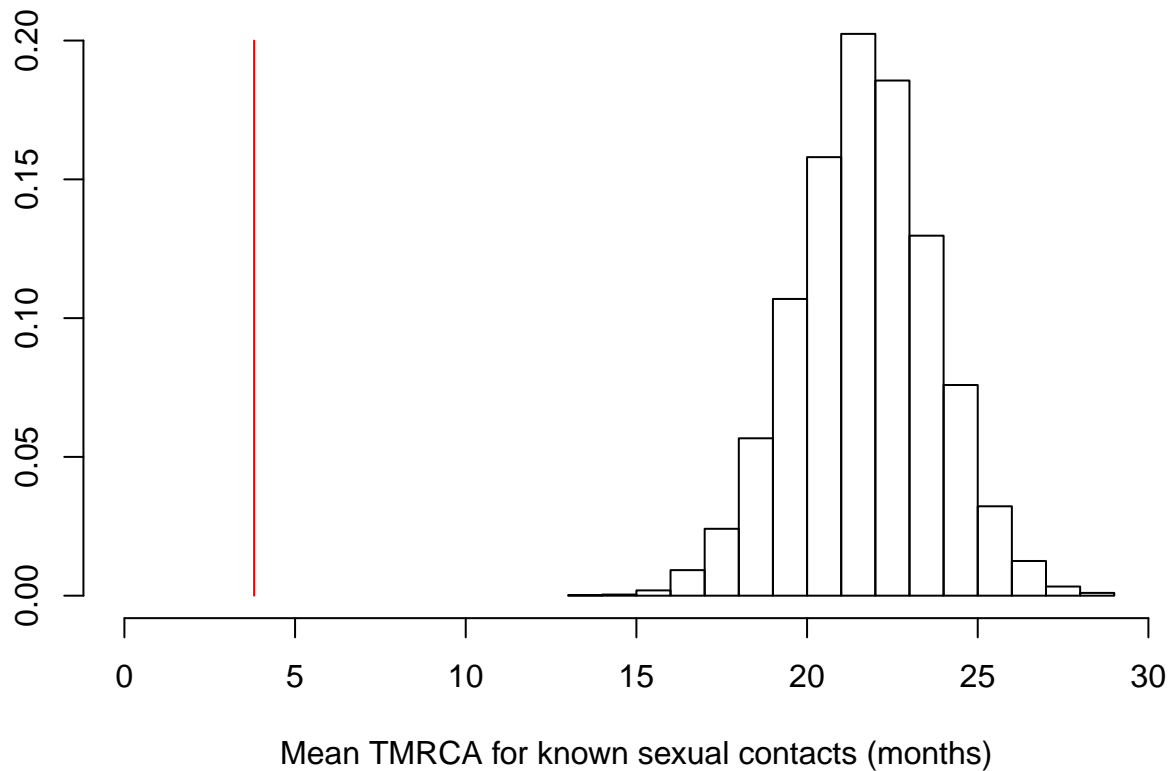

Supplement: Figure S2 — Permutation test comparing the mean TMRCA for known sexual contacts (in red) with values that would be obtained if the sexual contact labels were permuted at random (histogram). Download [file mbo003162861sf2.pdf]

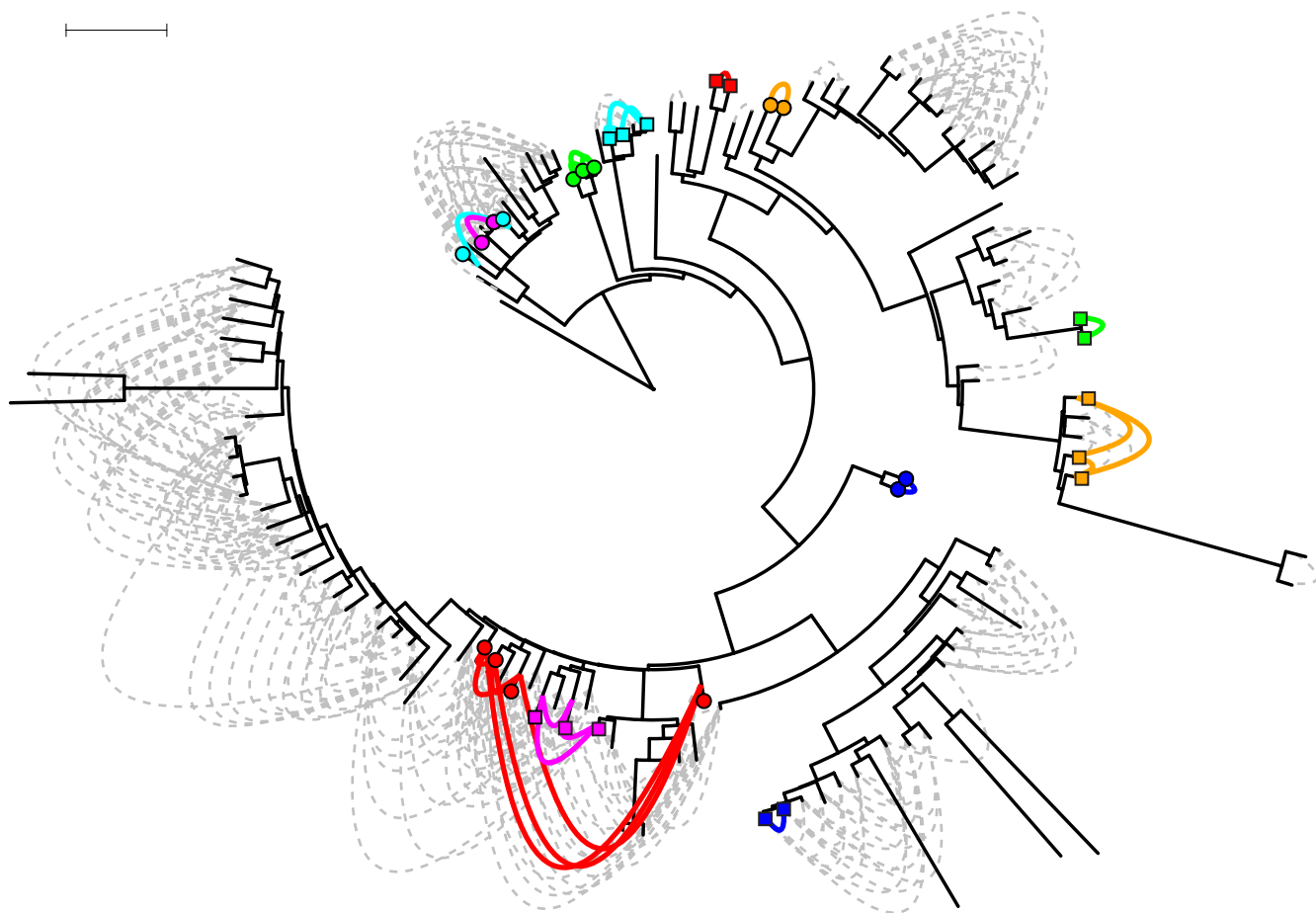

Supplement: Figure S3 — Links between the Sheffield genomes, based on the 8-month maximum TMRCA criterion. Download [file mbo003162861sf3.pdf]

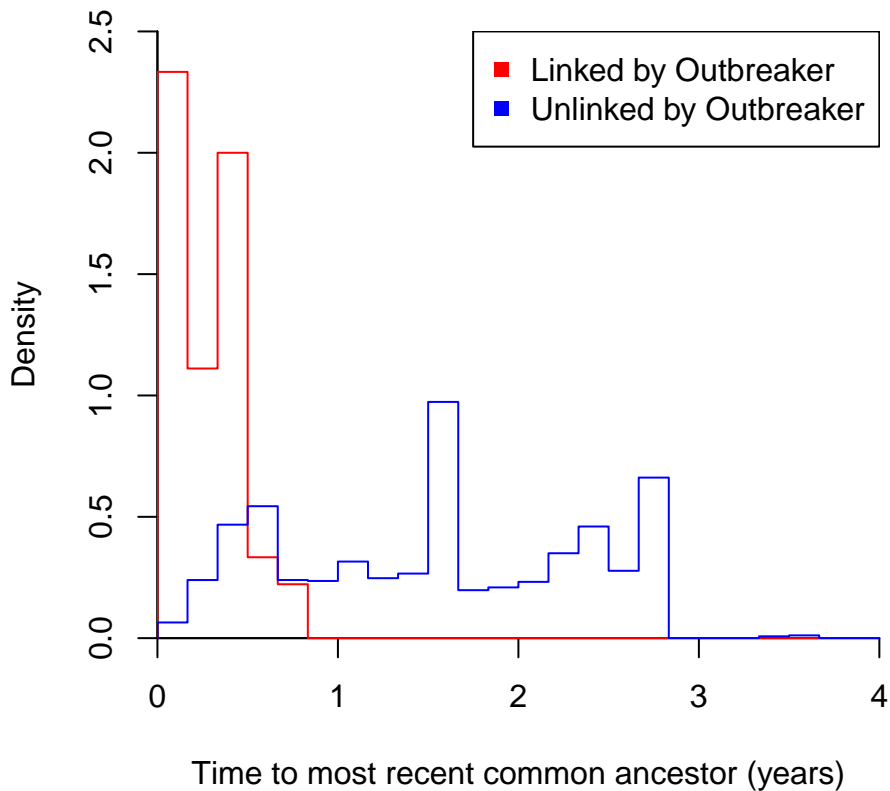

Supplement: Figure S4 — Distribution of TMRCA between pairs of genomes in the Sheffield data set that have been assessed to be directly linked by Outbreaker (red) or not (blue). Download [file mbo003162861sf4.pdf]

A

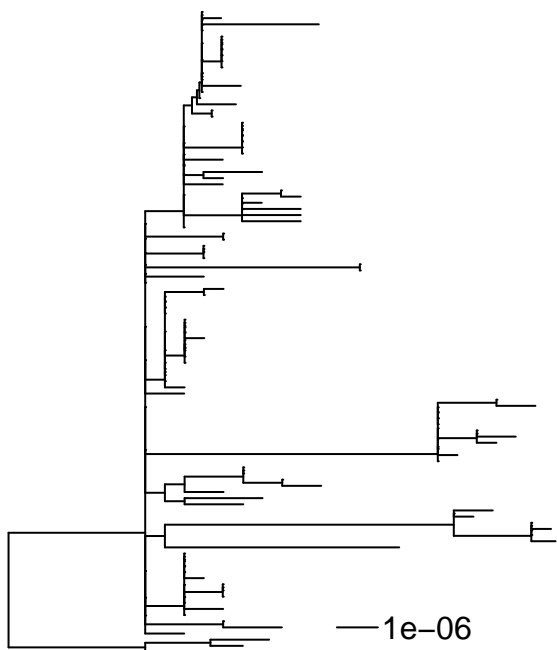

B

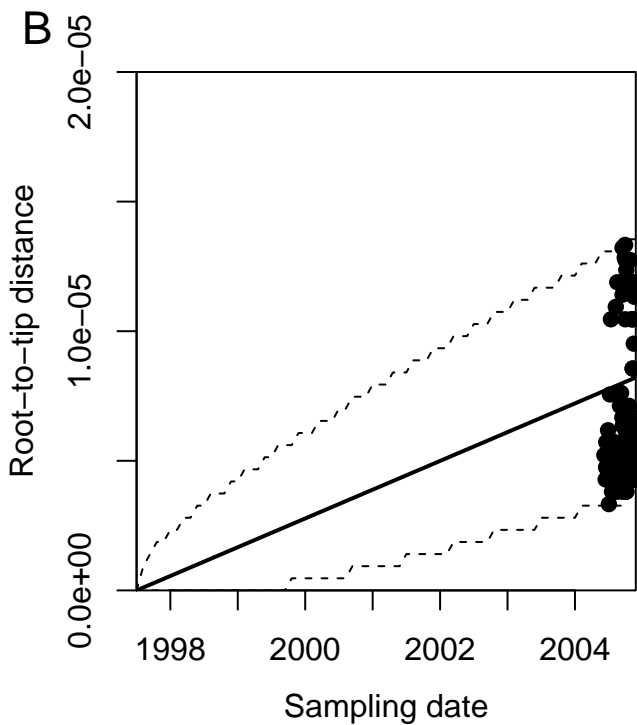

Supplement: Figure S5 — (A) Maximum likelihood tree reconstructed for the London genomes. (B) Temporal signal in the tree. The signal was not strong enough to estimate the evolutionary rate, and instead the solid line represents the rate estimated in Fig. S1 based on the Sheffield data. Download [file mbo003162861sf5.pdf]

I

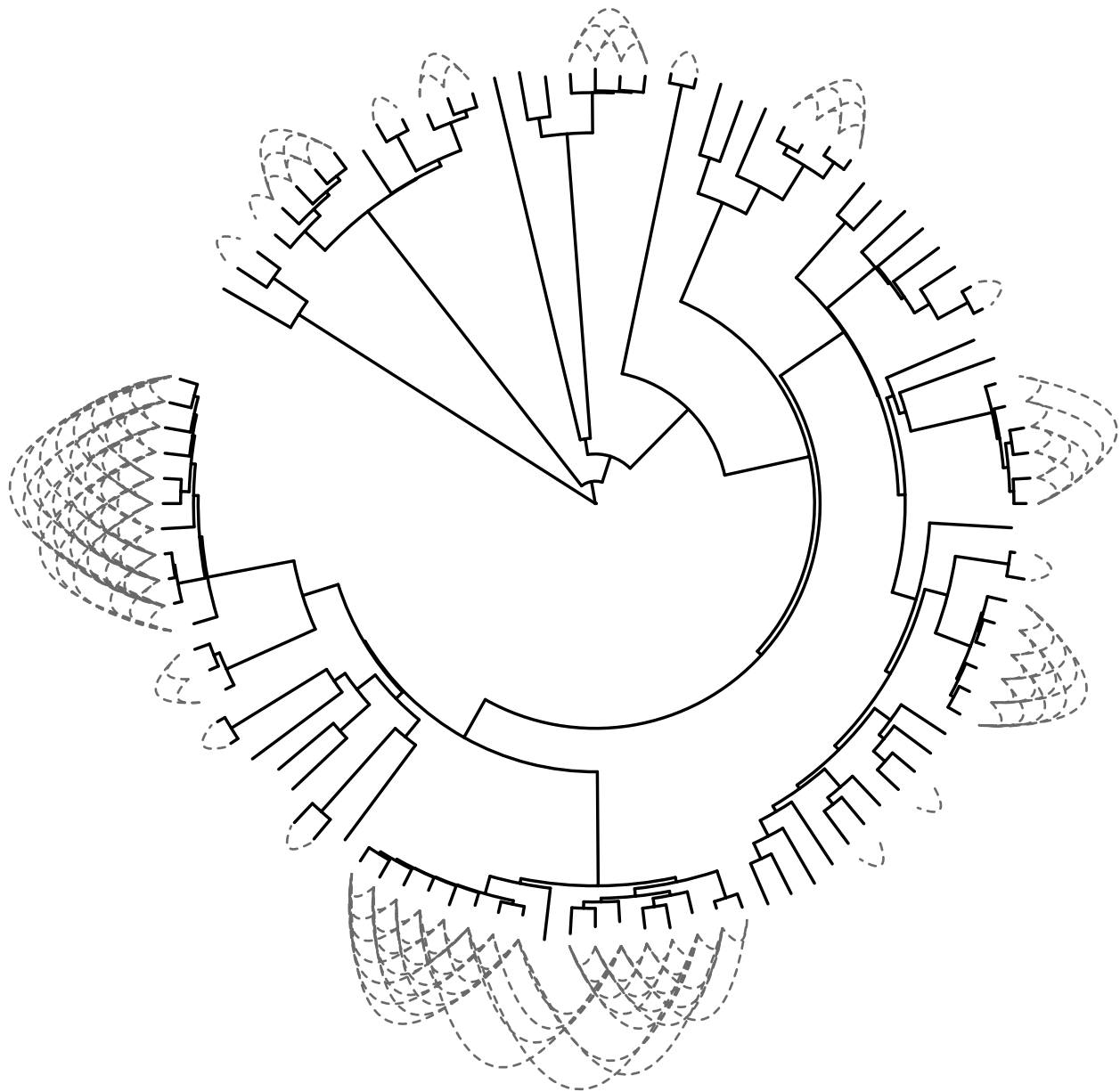

Supplement: Figure S6 — Links between the London genomes, based on the 8-month maximum TMRCA criterion. Download [file mbo003162861sf6.pdf]
