# Supplementary material for: Genomic Analysis and Comparison of Two Gonorrhea Outbreaks
Source: mBio. 2016 Jun 28;7(3):e00525-16. doi: 10.1128/mBio.00525-16 (PMC4937209; doi:10.1128/mBio.00525-16)
Supplement: Table S1 — List of 132 genomes in the Sheffield data set. [file mbo003162861st1.pdf]

isolate	Date.of.isolation	No.Contigs	Total.Length	Tracing.information	Sanger.ID	Accession number
S073	20/10/1995	75	2111762	Triplet S1	5505_5#3	ERR029245
S058	14/10/1995	78	2113385	Triplet S1	5505_5#2	ERR029244
S081	09/11/1995	81	2113731	Triplet S1	5505_5#4	ERR029246
S079	08/11/1995	81	2112082	-	6649_4#10	ERR049025
S151	24/02/1996	79	2115765	Pair S1	5505_5#9	ERR029251
S155	29/02/1996	82	2108072	Pair S1	5505_5#10	ERR029241
S349	24/03/1997	90	2103094	-	6649_5#11	ERR049047
S427	06/10/1997	76	2113738	-	5505_7#9	ERR029276
S491	18/11/1997	94	2102613	-	6649_6#13	ERR049070
NS008	30/06/1999	88	2105182	-	6649_4#1	ERR049016
S464	17/11/1997	83	2109212	-	6649_6#8	ERR049065
S522	11/12/1997	96	2114222	Pair 6	5314_3#10	ERR026601
S517	03/12/1997	116	2113638	Pair 6	5314_3#9	ERR026609
S526	23/12/1997	85	2105282	-	6649_6#21	ERR049078
S515	21/11/1997	78	2109105	-	6649_6#18	ERR049075
S414	10/06/1997	77	2110058	-	6648_1#10	ERR049004
S492	19/11/1997	85	2110479	-	6649_6#14	ERR049071
S446	16/12/1997	91	2111540	-	6649_6#2	ERR049059
NS028	08/09/1999	97	2103051	-	6649_4#2	ERR049017
NS056	01/12/1999	92	2109618	-	6649_4#5	ERR049020
S417	08/07/1997	78	2113283	-	6648_1#13	ERR049007
S382	23/06/1997	70	2116834	-	6648_1#3	ERR048997
S465	17/11/1997	94	2102252	-	6649_6#9	ERR049066
S373	13/05/1997	88	2111770	-	6649_5#20	ERR049056
S366	25/04/1997	85	2102932	-	6649_5#18	ERR049054
S368	28/04/1997	83	2110442	-	6649_5#19	ERR049055
S283	10/10/1996	90	2112582	-	5505_6#10	ERR029254
S255	23/08/1996	87	2112439	Quadruple S1	5505_6#5	ERR029260
S352	29/03/1997	82	2103588	-	6649_5#12	ERR049048
S360	09/04/1997	78	2110804	-	6649_5#15	ERR049051
S478	03/12/1997	80	2107795	-	6649_6#10	ERR049067
S474	17/12/1997	76	2111515	-	5505_7#10	ERR029267
S353	29/03/1997	83	2112608	-	6649_5#13	ERR049049
S306	10/12/1996	95	2110090	Triplet 2	5314_3#1	ERR026600
S330	11/02/1997	88	2104244	-	6649_5#7	ERR049043
S281	08/10/1996	77	2113974	-	5505_6#9	ERR029264
S317	02/01/1997	89	2122882	Triplet 2	5314_3#3	ERR026603
S308	04/01/1997	98	2119518	Triplet 2	5314_3#2	ERR026602
S302	05/11/1996	75	2108045	-	5505_7#4	ERR029271
S288	15/10/1996	84	2107087	Quadruple S1	5505_6#11	ERR029255
S290	02/11/1996	78	2129751	-	5505_7#1	ERR029266
S292	05/11/1996	77	2111586	Quadruple S1	5505_7#2	ERR029269
S278	03/10/1996	80	2112258	Quadruple S1	5505_6#7	ERR029262
S293	05/11/1996	79	2112521	-	5505_7#3	ERR029270
S387	04/07/1997	81	2126134	-	5505_7#8	ERR029275
S361	17/04/1997	83	2111321	-	6649_5#16	ERR049052
S380	26/04/1997	73	2115493	-	6648_1#2	ERR048996
S364	24/04/1997	91	2106443	-	6649_5#17	ERR049053
S416	27/06/1997	75	2115162	-	6648_1#12	ERR049006
S399	08/08/1997	79	2114559	-	6648_1#5	ERR048999
S509	23/10/1997	89	2106845	-	6649_6#16	ERR049073
S453	16/10/1997	84	2105740	-	6649_6#5	ERR049062
S481	16/10/1997	92	2104773	-	6649_6#11	ERR049068
S433	07/10/1997	75	2118087	-	6648_1#20	ERR049014
S418	08/08/1997	77	2114367	-	6648_1#14	ERR049008
S405	30/07/1997	107	2095201	-	6648_1#7	ERR049001
S449	01/08/1997	98	2099851	-	6649_6#4	ERR049061
NS031	27/09/1999	81	2103466	-	6649_4#3	ERR049018
NS055	01/12/1999	72	2111705	-	6649_4#4	ERR049019
S438	11/11/1997	80	2111745	-	6648_1#21	ERR049015
S488	11/11/1997	74	2110505	-	6649_6#12	ERR049069
S458	29/10/1997	89	2111237	-	6649_6#6	ERR049063
S461	10/11/1997	92	2104525	-	6649_6#7	ERR049064
S511	07/11/1997	105	2098748	-	6649_6#17	ERR049074
S406	25/09/1997	73	2118126	-	6648_1#8	ERR049002
S415	27/06/1997	82	2110377	-	6648_1#11	ERR049005
S339	10/01/1997	100	2097288	-	6649_5#9	ERR049045
S346	24/02/1997	93	2101432	-	6649_5#10	ERR049046
S320	05/02/1997	90	2109951	-	6649_5#5	ERR049041
S311	13/01/1997	88	2104304	-	6649_5#2	ERR049038
S378	09/05/1997	73	2115886	-	6648_1#1	ERR048995
S276	27/09/1996	78	2131389	-	5505_6#6	ERR029261
S419	13/06/1997	79	2115592	-	6648_1#15	ERR049009
S358	08/04/1997	90	2106271	-	6649_5#14	ERR049050
S350	08/04/1997	78	2116621	-	5505_7#6	ERR029273
S359	04/08/1997	81	2109899	-	5505_7#7	ERR029274
S310	06/01/1997	84	2106568	-	6649_5#1	ERR049037
S377	13/05/1997	89	2104899	-	6649_5#21	ERR049057
S514	21/11/1997	100	2113691	Pair 5	5314_3#8	ERR026608
S493	20/11/1997	118	2110210	Pair 5	5314_3#7	ERR026607
S343	18/02/1997	79	2111201	-	5505_7#5	ERR029272
S280	07/10/1996	81	2116936	-	5505_6#8	ERR029263
S508	22/10/1997	85	2117400	-	6649_6#15	ERR049072
S524	13/11/1997	90	2116505	-	6649_6#19	ERR049076
S463	11/11/1997	109	2114785	Triplet 3	5314_3#5	ERR026605
S445	07/11/1997	100	2119392	Triplet 3	5314_3#4	ERR026604
S466	19/11/1997	111	2116890	Triplet 3	5314_3#6	ERR026606
NS101	02/03/2000	84	2114561	-	6649_4#6	ERR049021
NS105	22/03/2000	89	2106632	-	6649_4#7	ERR049022
S289	07/10/1996	75	2110594	-	5505_6#12	ERR029256
S424	08/08/1997	79	2114635	-	6648_1#17	ERR049011
S423	08/07/1997	76	2113662	-	6648_1#16	ERR049010
S397	18/07/1997	75	2116799	-	6648_1#4	ERR048998
S448	19/06/1997	84	2108932	-	6649_6#3	ERR049060
S407	25/06/1997	79	2111020	-	6648_1#9	ERR049003
S401	17/09/1997	82	2115212	-	6648_1#6	ERR049000
S525	26/09/1997	91	2102925	-	6649_6#20	ERR049077
S441	01/08/1997	87	2106987	-	6649_6#1	ERR049058
S430	02/07/1997	74	2119138	-	6648_1#19	ERR049013
S425	01/08/1997	79	2114458	-	6648_1#18	ERR049012
S329	07/02/1997	92	2104782	-	6649_5#6	ERR049042
S319	04/02/1997	91	2113179	-	6649_5#4	ERR049040
S316	18/12/1996	82	2112298	-	6649_5#3	ERR049039
S256	24/08/1996	103	2117616	Pair 3	5314_2#10	ERR026589
S241	14/08/1996	99	2115158	Pair 3	5314_2#8	ERR026597
S217	18/06/1996	76	2116203	-	5505_6#4	ERR029259
S184	18/04/1996	82	2117998	-	5505_5#12	ERR029243
S257	24/08/1996	105	2112756	Pair 4	5314_2#11	ERR026590
S253	23/08/1996	105	2113861	Pair 4	5314_2#9	ERR026598
S203	15/05/1996	94	2105176	-	6649_4#17	ERR049032
S207	22/05/1996	85	2117040	-	5505_6#3	ERR029258
S206	20/05/1996	84	2115544	-	5505_6#2	ERR029257
S153a	28/02/1996	80	2119409	-	6649_4#15	ERR049030
S152	28/02/1996	94	2113575	Triplet1	5314_2#6	ERR026595
S154	29/02/1996	89	2117703	Triplet1	5314_2#7	ERR026596
S158	29/02/1996	83	2115290	-	6649_4#16	ERR049031
S149	23/02/1996	92	2117858	Triplet1	5314_2#5	ERR026594
S098	09/11/1995	92	2115728	Pair 2	5314_2#3	ERR026592
S100	24/11/1995	106	2111754	Pair 2	5314_2#4	ERR026593
S092	20/11/1995	91	2111791	-	6649_4#11	ERR049026
S106	01/12/1995	77	2120409	-	5505_5#5	ERR029247
S201	14/05/1996	80	2116178	-	5505_6#1	ERR029253
S163	09/03/1996	87	2114870	-	5505_5#11	ERR029242
S141	06/02/1996	82	2113214	-	5505_5#8	ERR029250
S142	06/02/1996	82	2110833	-	6649_4#14	ERR049029
S135	25/01/1996	84	2106984	-	5505_5#7	ERR029249
S105	01/12/1995	91	2100990	-	6649_4#13	ERR049028
S078	20/10/1995	104	2115489	Pair 1	5314_2#1	ERR026588
S096	22/11/1995	88	2109963	-	6649_4#12	ERR049027
S086	09/11/1995	98	2112046	Pair 1	5314_2#2	ERR026591
S041	08/08/1995	94	2105583	-	6649_4#8	ERR049023
S006	11/04/1995	77	2113804	-	5505_5#1	ERR029240
