# Supplementary material for: Genomic Analysis and Comparison of Two Gonorrhea Outbreaks
Source: mBio. 2016 Jun 28;7(3):e00525-16. doi: 10.1128/mBio.00525-16 (PMC4937209; doi:10.1128/mBio.00525-16)
Supplement: Table S2 — List of 105 genomes in the London data set. [file mbo003162861st2.pdf]

strain	date_of_isolation	gender	Sexual_Orientation	HIV_Status	Year_of_birth	Ethnicity	OS_Partners	UK_Partners	Previous_Gonorrhoea	postcode	Sanger.ID	Accession number
LG1291	27/09/2004	M	Homosexual	HIV Negative	1981	White British	0	2	N	SE25	8987_7#5	ERR234417
LG1834	17/11/2004	M	Bisexual	Unknown	1976	White British	0	2	N		8987_7#87	ERR234499
W0603	01/10/2004	M	Homosexual	Unknown	1978	White British	NA	1	U	SE38	8987_6#63	ERR234397
W0561	15/09/2004	M	Homosexual	Unknown	1981	Other Asian	2	3	N	SE14	8987_6#57	ERR234391
W0789	28/10/2004	M	Homosexual	HIV Positive	1981	White British	0	1	N	E151	8987_6#75	ERR234409
W0613	29/09/2004	M	Homosexual	Unknown	1981	Other Asian	2	3	N	SE14	8987_6#66	ERR234400
LG1721	15/11/2004	M	Homosexual	Unknown	1960	Other white	0	2	Y	SW90	8987_7#7	ERR234419
LG0261	28/06/2004	M	Homosexual	HIV Negative	1985	Other white	0	1	Y		8987_7#41	ERR234453
LG0394	12/07/2004	M	Homosexual	HIV Negative	1963	White British	1	0	N	NW6	8987_7#2	ERR234414
W0185	12/07/2004	M	Homosexual	HIV Negative	1977	Other white	0	2	N	N15	8987_7#40	ERR234452
LG0660	02/08/2004	M	Homosexual	HIV Positive	1952	White British	2	8	Y	WC1H	8987_7#90	ERR234502
LG1349	06/10/2004	M	Homosexual	HIV Positive	1976	Other white	0	3	N		8987_7#45	ERR234457
LG1636	04/11/2004	M	Homosexual	HIV Negative	1957	White British	0	4	Y	SE37	8987_7#62	ERR234474
LG1635	04/11/2004	M	Homosexual	HIV Negative	1957	White British	0	4	Y	SE37	8987_7#54	ERR234466
LG1628	01/11/2004	M	Homosexual	HIV Negative	1969	Other white	0	2	Y	E17	8987_7#38	ERR234450
LG0931	24/08/2004	M	Homosexual	HIV Negative	1969	White British	0	1	Y	N28H	8987_7#75	ERR234487
W0065	08/06/2004	M	Homosexual	HIV Negative	1980	White Irish	1	1	Y	N227	8987_7#32	ERR234444
LG1375	08/10/2004	F	Heterosexual	HIV Negative	1974	Other white	1	0	N	N165	8987_7#61	ERR234473
LG1861	23/11/2004	M	Homosexual	HIV Negative	1986	White British	0	0	N	SM67	8987_7#16	ERR234428
LG1274	07/11/2004	M	Homosexual	HIV Negative	1982	Other Ethnic Group	9	0	Y	W2	8987_7#84	ERR234496
LG1781	10/11/2004	M	Homosexual	HIV Negative	1982	White British	0	1	N	SE183	8987_7#63	ERR234475
LG0910	20/08/2004	M	Homosexual	HIV Negative	1982	Other Ethnic Group	9	0	Y	W2	8987_7#67	ERR234479
LG0458	14/07/2004	M	Heterosexual	HIV Negative	1982	White British	NA	1	Y	SE10	8987_7#10	ERR234422
LG0273	22/06/2004	M	Homosexual	HIV Negative	1977	White British	NA	3	N	NW2 4	8987_7#49	ERR234461
LG0516	19/07/2004	F	Heterosexual	Unknown	1978	White British	0	1	N	SE12	8987_7#42	ERR234454
LG0355	06/07/2004	M	Homosexual	HIV Negative	1982	Other Ethnic Group	2	0	Y	SW2	8987_7#81	ERR234493
LG0353	06/07/2004	M	Homosexual	HIV Negative	1982	Other Ethnic Group	2	0	Y	SW2	8987_7#73	ERR234485
W0251	15/07/2004	M	Homosexual	HIV Negative	1974	Other Black	0	1	N	WC2P	8987_7#64	ERR234476
LG1644	04/11/2004	M	Homosexual	HIV Negative	1981	White British	0	2	Y	E15	8987_7#70	ERR234482
LG0790	12/08/2004	M	Homosexual	Unknown	1982	White British	0	2	Y	ME194	8987_7#51	ERR234463
LG0675	04/08/2004	M	Homosexual	HIV Negative	1982	White British	NA	3	N	NW1	8987_7#19	ERR234431
W0239	20/07/2004	M	Homosexual	HIV Negative	1971	White British	NA	2	N		8987_7#56	ERR234468
LG0285	25/06/2004	M	Homosexual	HIV Positive	1973	White British	NA	6	Y	SE114	8987_7#57	ERR234469
LG0735	09/08/2004	M	Homosexual	Unknown	1980	White Irish	0	2	N	SE1	8987_7#35	ERR234447
LG0211	18/06/2004	M	Homosexual	HIV Positive	1966	White British	NA	100	Y	E15	8987_7#25	ERR234437
W0204	06/07/2004	M	Homosexual	HIV Negative	1963	Other Black	1	2	Y	SE12	8987_7#48	ERR234460
LG0383	08/07/2004	M	Homosexual	HIV Positive	1983	Other Ethnic Group	NA	1	N	SW9 9	8987_7#89	ERR234501
W0604	01/10/2004	M	Homosexual	Unknown	1969	White British	NA	2	U	SE11	8987_6#64	ERR234398
W0761	21/10/2004	M	Homosexual	Unknown	1971	White Irish	NA	1	N	SE15	8987_6#72	ERR234406
W0751	21/10/2004	M	Homosexual	HIV Negative	1961	White British	NA	1	N	N1 8	8987_6#71	ERR234405
W0587	17/09/2004	M	Homosexual	HIV Negative	1970	Other white	0	2	N	EC1V	8987_6#61	ERR234395
W0563	13/09/2004	M	Homosexual	Unknown	1957	White British	0	2	Y	SW83	8987_6#58	ERR234392
W0420	01/09/2004	M	Homosexual	Unknown	1967	White British	0	1	N	N88	8987_6#53	ERR234387
LG1850	26/11/2004	M	Homosexual	HIV Negative	1977	Unknown	0	2	Y	N15 6	8987_7#95	ERR234507
W0264	23/07/2004	M	Homosexual	HIV Positive	1973	White British	0	2	Y	E7	8987_7#80	ERR234492
LG1133	15/09/2004	M	Homosexual	HIV Positive	1977	Other white	0	15	N	N19	8987_7#44	ERR234456
LG1365	08/10/2004	M	Homosexual	HIV Negative	1970	Other white	0	20	Y	E2 6	8987_7#53	ERR234465
LG1091	09/09/2004	M	Homosexual	Unknown	1972	Other white	0	1	N	SW73	8987_7#4	ERR234416
LG0671	04/08/2004	M	Homosexual	HIV Positive	1960	Other white	0	2	N	IG26	8987_7#3	ERR234415
LG1094	10/09/2004	M	Homosexual	HIV Positive	1965	White British	NA	5	Y	W86J	8987_7#12	ERR234424
LG0621	29/07/2004	M	Homosexual	HIV Negative	1971	White British	0	1	N	NW86	8987_7#66	ERR234478
LG0465	13/07/2004	M	Homosexual	HIV Positive	1969	Other white	0	8	Y	E2	8987_7#26	ERR234438
W0362	12/08/2004	M	Homosexual	HIV Negative	1978	Unknown	0	2	Y	EC1A	8987_6#51	ERR234385
W0569	27/09/2004	M	Heterosexual	Unknown	1962	Black African	0	3	Y	SE16	8987_6#60	ERR234394
LG1346	05/10/2004	M	Homosexual	HIV Negative	1973	White British	0	3	N		8987_7#29	ERR234441
LG0795	16/08/2004	M	Homosexual	HIV Negative	1976	Other white	0	2	N	NW10 5	8987_7#59	ERR234471
LG0493	19/07/2004	M	Homosexual	HIV Negative	1964	White British	0	2	Y		8987_7#34	ERR234446
LG1126	13/09/2004	M	Homosexual	HIV Positive	1980	White British	2	3	Y	E83H	8987_7#28	ERR234440
LG1342	04/10/2004	M	Heterosexual	Unknown	1963	White British	1	1	N	SW12	8987_7#21	ERR234433
W0296	30/07/2004	M	Homosexual	Unknown	1974	Other Ethnic Group	0	3	U	SE165	8987_7#88	ERR234500
LG0989	06/09/2004	M	Bisexual	HIV Negative	1979	Other Ethnic Group	NA	NA	N	SW18	8987_7#83	ERR234495
LG0640	28/07/2004	M	Homosexual	Unknown	1975	White Irish	NA	3	N	E11	8987_7#82	ERR234494
LG0205	17/06/2004	M	Homosexual	Unknown	1960	Other Asian	2	6	N	SW7	8987_7#17	ERR234429
W0725	21/10/2004	M	Homosexual	Unknown	1971	White British	0	1	Y		8987_6#69	ERR234403
W0762	18/10/2004	M	Homosexual	HIV Positive	1959	White British	NA	4	Y	SE18	8987_6#73	ERR234407
W0749	18/10/2004	M	Bisexual	Unknown	1978	White British	NA	2	N	E1 8	8987_6#70	ERR234404
LG1423	11/10/2004	M	Homosexual	Unknown	1962	White British	1	2	N	NW3	8987_7#69	ERR234481
LG1477	14/10/2004	M	Homosexual	HIV Positive	1970	White British	0	5	Y	NW1	8987_7#85	ERR234497
LG1548	22/10/2004	M	Homosexual	HIV Positive	1961	Other white	NA	2	Y	SW84	8987_7#14	ERR234426
LG1810	16/11/2004	M	Homosexual	HIV Positive	1964	White British	0	4	N	N1	8987_7#79	ERR234491
LG1745	12/11/2004	M	Homosexual	Unknown	1971	Other white	NA	4	Y	SE26	8987_7#47	ERR234459
LG1580	21/10/2004	M	Homosexual	HIV Positive	1964	White British	NA	10	N	SW9	8987_7#22	ERR234434
LG1619	25/10/2004	M	Unknown	Unknown	1966	Other Asian	0	7	N	E3 4	8987_7#30	ERR234442
LG0541	22/07/2004	M	Homosexual	HIV Negative	1969	White British	1	1	N	RH1	8987_7#50	ERR234462
LG0186	15/06/2004	M	Homosexual	HIV Positive	1978	White British	0	1	N	SM	8987_7#1	ERR234413
LG0311	02/07/2004	M	Homosexual	Unknown	1941	White British	0	2	Y	SW18	8987_7#65	ERR234477
LG1132	14/09/2004	F	Heterosexual	Unknown	1953	White British	1	1	N	TQ6	8987_7#36	ERR234448
LG1739	10/11/2004	M	Homosexual	HIV Negative	1965	White British	NA	2	N	W14	8987_7#31	ERR234443
LG1740	10/11/2004	M	Homosexual	HIV Negative	1965	White British	NA	2	N	W14	8987_7#39	ERR234451
W0255	22/07/2004	M	Homosexual	HIV Negative	1978	Pakistani	NA	2	N	UB40	8987_7#72	ERR234484
LG1865	24/11/2004	M	Homosexual	Unknown	1969	White British	0	1	N	SG14	8987_7#24	ERR234436
W0885	19/11/2004	M	Homosexual	HIV Negative	1963	White British	NA	20	N	SE14	8987_6#77	ERR234411
LG1137	15/09/2004	M	Homosexual	Unknown	1970	Black Caribbean	4	6	U	SW52	8987_7#52	ERR234464
LG1799	18/11/2004	M	Homosexual	HIV Negative	1968	Other Ethnic Group	NA	1	N	SW1X	8987_7#71	ERR234483
LG1289	28/09/2004	M	Homosexual	HIV Negative	1982	White British	3	7	N	SW1V	8987_7#92	ERR234504
LG1197	14/09/2004	M	Bisexual	Unknown	1980	Indian	1	0	N	E130	8987_7#60	ERR234472
LG1114	07/09/2004	M	Bisexual	Unknown	1980	Indian	1	0	N	E130	8987_7#20	ERR234432
LG0199	18/06/2004	M	Homosexual	Unknown	1981	White British	0	4	N	SW20	8987_7#9	ERR234421
LG0633	02/08/2004	M	Homosexual	HIV Positive	1967	Other white	6	0	Y	W10	8987_7#74	ERR234486
LG1737	09/11/2004	M	Homosexual	HIV Negative	1975	White British	1	1	Y	EI75	8987_7#23	ERR234435
LG1736	09/11/2004	M	Homosexual	HIV Negative	1975	White British	1	1	Y	EI75	8987_7#15	ERR234427
W0592	27/09/2004	M	Homosexual	HIV Positive	1965	White British	NA	3	Y	N4 1	8987_6#62	ERR234396
LG1719	15/11/2004	M	Heterosexual	HIV Negative	1979	Other white	2	0	N	SE17	8987_7#94	ERR234506
W0560	15/09/2004	M	Homosexual	Unknown	1981	White British	0	1	N	SE13	8987_6#56	ERR234390
W0627	05/10/2004	M	Homosexual	HIV Positive	1966	White British	NA	4	U	E8 2	8987_6#68	ERR234402
W0370	17/08/2004	M	Homosexual	Unknown	1964	White British	0	2	Y	SE15	8987_6#52	ERR234386
LG0751	09/08/2004	M	Homosexual	HIV Negative	1969	Other white	0	2	Y	CR0 6	8987_7#43	ERR234455
LG0463	13/07/2004	M	Homosexual	HIV Positive	1967	Other white	NA	2	Y	N134	8987_7#18	ERR234430
LG1295	30/09/2004	M	Homosexual	HIV Negative	1977	Pakistani	NA	9	N	N8	8987_7#13	ERR234425
LG1757	12/11/2004	M	Homosexual	HIV Positive	1952	Other white	0	2	N	NW10 9	8987_7#55	ERR234467
LG1687	04/11/2004	M	Homosexual	HIV Negative	1972	White British	3	0	Y	SW10	8987_7#86	ERR234498
LG1630	04/11/2004	M	Homosexual	HIV Negative	1972	White British	3	0	Y	SW10	8987_7#46	ERR234458
LG1248	24/09/2004	M	Homosexual	HIV Negative	1970	Other Asian	0	1	N	HA3	8987_7#76	ERR234488
W0306	03/08/2004	M	Homosexual	HIV Positive	1959	White British	NA	1	Y	WC14	8987_6#49	ERR234383
W0550	16/09/2004	M	Homosexual	Unknown	1982	White British	0	1	N	W68L	8987_6#55	ERR234389
